# Evaluation of the Pericardium with CT and MR

**DOI:** 10.1155/2014/174908

**Published:** 2014-01-29

**Authors:** Julianna M. Czum, Anne M. Silas, Morgan C. Althoen

**Affiliations:** ^1^Dartmouth-Hitchcock Medical Center, One Medical Center Drive, Lebanon, NH 03756, USA; ^2^St. Luke's Hospital, 915 East First Street, Duluth, MN 55805, USA

## Abstract

The pericardium plays an important role in optimizing cardiac motion and chamber pressures and serves as a barrier to pathology. In addition to pericardial anatomy and function, this review article covers a variety of pericardial conditions, with mention of potential pitfalls encountered during interpretation of diagnostic imaging. Normal and abnormal appearance of pericardium on CT and MR imaging is emphasized, including dynamic imaging correlates of pericardial pathophysiology.

## 1. Introduction

More than just a tissue, the pericardium is an organ with specific functions and an embryologic origin distinct from the heart. Whereas the heart is derived from splanchnic mesoderm, the pericardium is derived from somatic mesoderm [1–3]. Long-recognized functions of the pericardium include anchoring the heart in the mediastinum, minimizing the friction of cardiac motion, and serving as a barrier from infection and neoplasm [4]. More recently, the pericardium has been described as an intracardiac pressure modulator, limiting acute distention of any one cardiac chamber and preserving myofibril function by preventing sarcomere overlengthening [5, 6].

As with other organs, the pericardium is subject to various disease processes, include inflammatory, infectious, fibrotic, metabolic, and neoplastic. Imaging of these processes has advanced significantly in the past decade, with the refinement of multidetector CT and high-field-strength MRI. CT and MR permit visualization of the entire pericardium by virtue of three-dimensional acquisition and multiplanar imaging, respectively, and provide better assessment of surrounding structures than the prior standard of echocardiography [7]. In addition, MR techniques allow the evaluation of pericardial function, particularly as it relates to the problem of differentiating myocardial restriction from pericardial constriction, the latter being surgically treatable [8].

## 2. Anatomic Considerations

**Figure 1:**
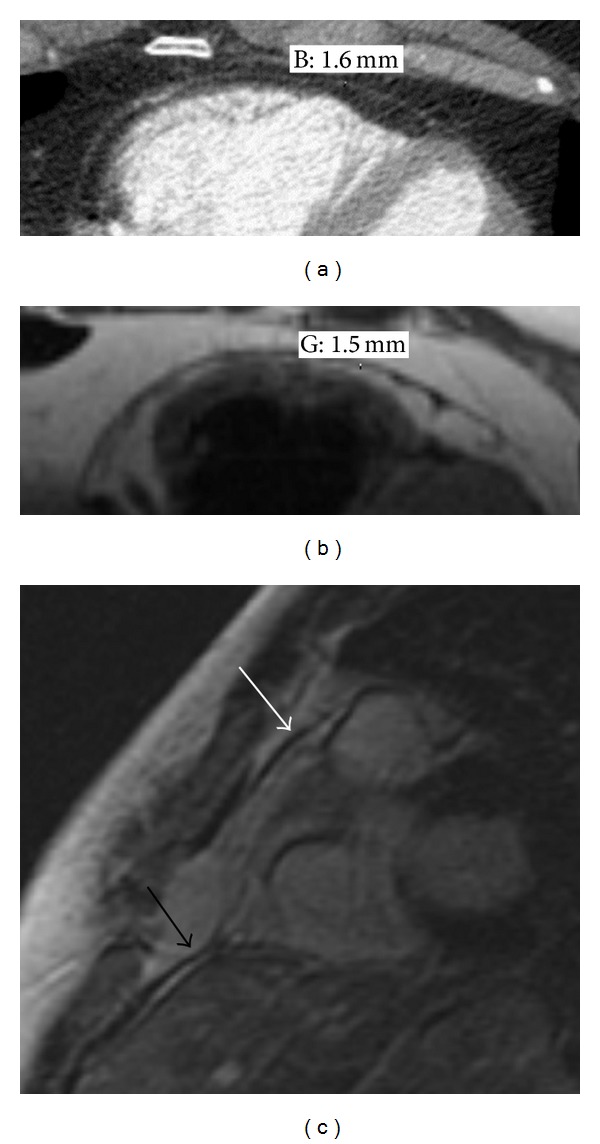
Normal pericardium. (a) Gated contrast-enhanced axial CT and (b) axial double inversion recovery MR images from the same patient show the normal thickness pericardium (parietal and visceral layers indistinguishable) sandwiched between epicardial and pericardial fat layers. (c) A sagittal postcontrast gradient MR image demonstrates both pericardial-diaphragmatic (black arrow) and pericardial-sternal (white arrow) ligaments.

As with the other serosal surfaces of the body, the pericardium has parietal and visceral layers. The parietal layer of pericardium is several times thicker than the visceral pericardium [4]. The normal combined pericardial thickness is 2 mm or less (Figures 1(a) and 1(b)). 2-3 mm is considered equivocal, whereas 4 mm thickness at any point is abnormal [9, 10]. The normal pericardial stabilizers include the great vessel reflections and several ligaments (pericardial-sternal, pericardial-vertebral, and pericardial-diaphragmatic) (Figure 1(c)) [4].

**Figure 2:**
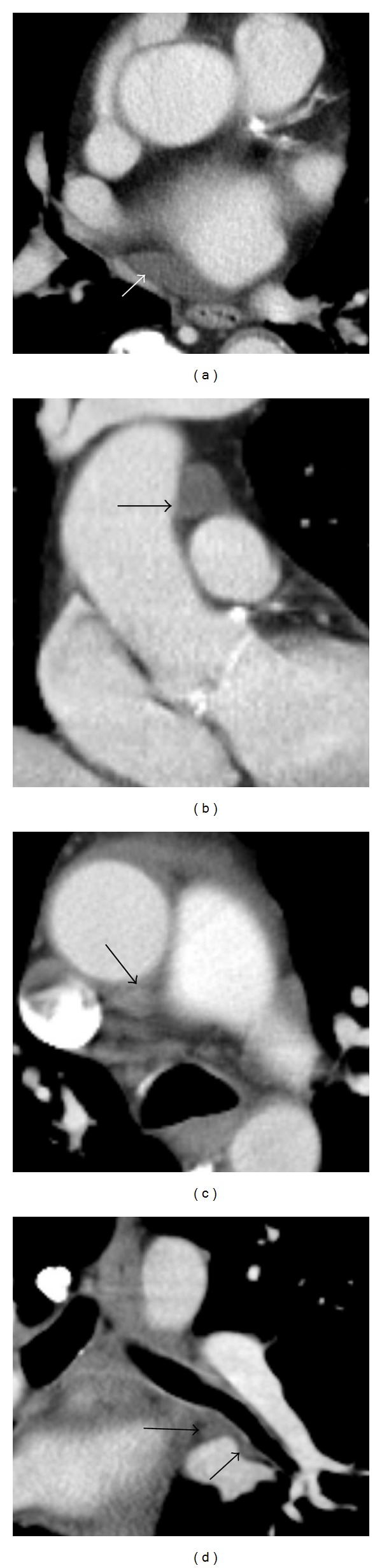
Normal pericardial recesses may be confused with pathology. (a) Contrast-enhanced CT (CECT) demonstrates fluid in the oblique sinus (white arrow). (b, c) Fluid in the superior aortic recess (black arrows). This may appear dense on CT due to contrast blooming and maybe mistaken for soft tissue. (d) Fluid in the left inferior pulmonary venous recess (black arrows).

Normal pericardial recesses occur due to the closer apposition of the visceral layer than the parietal layer to the contours of the heart and great vessels. Also, portions of the left atrium are left uncovered by pericardium to a variable degree [11]. These factors result in fluid-filled normal spaces which can be mistaken for pathology [12, 13]. For example, the oblique sinus may simulate an esophageal lesion or paraesophageal lymph node (Figure 2(a)). The normal superior aortic recess may also simulate soft tissue, particularly a mediastinal lymph node, due to blooming artifact caused by intravenous contrast in the adjacent great vessels (Figures 2(b) and 2(c)). The normal pericardial space contains 15–50 mL of fluid, an ultrafiltrate of plasma [8]. Much of this fluid is contained within normal but variable pericardial recesses (Figure 2(d)) [14].

## 3. Imaging Techniques

On a scale from 1 to 9, 1 being the least appropriate and 9 being most appropriate, both CT and MR are assigned a score of 8 for the evaluation of pericardial disease (per a multisociety consensus statement) [7, 15].

### 3.1. CT

ECG-gated multidetector row CT is useful for pericardial imaging, with a minimum of 16 detector rows, but preferably 64 or higher. If inflammatory, infectious, or neoplastic etiologies are considered, delayed imaging after intravenous contrast administration is preferred over first-pass cardiac imaging to permit the contrast bolus to clear the great vessels. This reduces blooming and streak artifacts and allows time for inflamed or neoplastic tissues to optimally enhance.

### 3.2. MR

Like echocardiography, MR can demonstrate morphology and function. MR affords advantages such as better tissue characterization, visualization of adjacent noncardiac structures, and lack of acoustic window constraints, allowing the entire pericardium to be imaged (Table 1) [16]. MR easily images the normal pericardium as a thin hypointense band sandwiched between the layers of epicardial and pericardial fat [17]. Common indications for pericardial imaging with MR are: distinguishing constrictive pericarditis from restrictive cardiomyopathy, distinguishing infectious pericarditis from myocarditis, and evaluating for pericardial neoplasm.

## 4. Effusion and Tamponade

As pericardial fluid volume is not easily measured, pericardial effusion can be defined as separation of the parietal and visceral layers by a sufficient amount of fluid to be detected on imaging (excluding the normal pericardial recesses). The differential diagnosis of pericardial effusion is extensive (Table 2) but can often be narrowed depending on the clinical situation of the patient. For example, pericardial effusion in the setting of rheumatoid arthritis, congestive heart failure, and metastatic malignancy is commonly attributed to the underlying disease [18]. Malignant pericardial effusion is usually accompanied by irregular pericardial thickening and enhancement and frequently mediastinal lymphadenopathy [19]. Cardiac imagers using CT and MR are asked to assess effusion size, location, acuity, composition (simple or complex), etiology, impaired remodeling, and hemodynamic significance.

In the case of physiologically significant pericardial effusions, the absolute volume is less important than rate of fluid volume accumulation. Only 150–250 mL of pericardial sac fluid is needed to cause tamponade acutely, whereas slow accumulations, such as in thyroid myxedema, can reach 3 L without tamponade, as the pericardium will remodel over time [18, 20, 21].

Normally, intrathoracic pressure changes are transmitted through the pericardium to the cardiac chambers, with respirophasic influence upon systemic venous return and right ventricular filling. In addition, the right and left ventricles are affected by pressure differences between them transmitted across the interventricular septum, normally higher on the left, with convex border of the septum relative to the right ventricle chamber. In the setting of tamponade, the cardiac chamber pressure differences are diminishingly influenced by intrathoracic pressure changes with respiration, leaving transseptal pressure differences to exert their effects upon the cardiac chambers, a phenomenon known as ventricular coupling or ventricular interdependence [4, 22].

**Figure 3:**
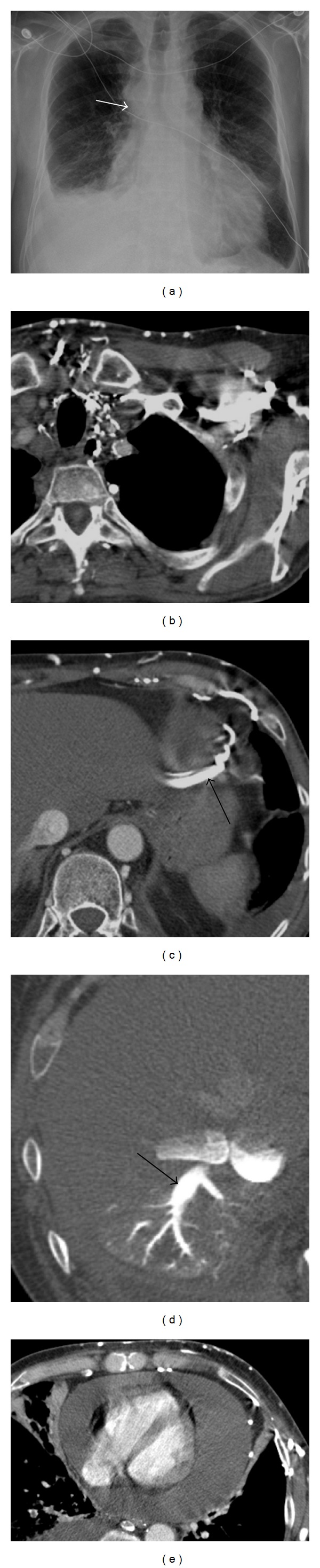
A 55-year-old previously healthy male with dyspnea. (a) Chest radiograph shows an enlarged cardiac silhouette, a right pleural effusion, and dilated azygos vein (arrow), (b) CECT demonstrates extensive venous collaterals around the heart, including prominent filling of (b) subcutaneous, (c) inferior phrenic, and (d) hepatic veins. (e) CECT shows a large pericardial effusion. The interventricular septum is flattened. The constellation of effusion, flat septum, and impaired venous return (b–d) is consistent with tamponade physiology. The patient's symptoms and blood pressure improved after pericardiocentesis. Fluid cytology was positive for malignant cells. He was later diagnosed with nonsmall cell lung cancer.

On dynamic imaging, ventricular interdependence is manifested by rocking motion of the interventricular septum during the cardiac cycle. Specifically, the septum moves toward the left ventricle in early diastole as the right ventricle fills with systemic blood resulting in a transient relative elevation of right heart pressure. The septum moves back toward the right ventricle only in late diastole as the pressure on the left eventually exceeds that of the right. With prolonged or severe tamponade, right ventricular filling becomes impaired as right ventricular end-diastolic pressure (and right atrial pressure) approaches central venous pressure. On imaging, this can be suspected if the contrast bolus refluxes into dilated hepatic veins or collateral vessels (Figure 3) [23]. The end-stage occurs with systemic and pulmonary venous pooling, resulting in equalization of chamber pressures and complete left ventricular diastolic failure [4, 20].

## 5. Constriction

**Figure 4:**
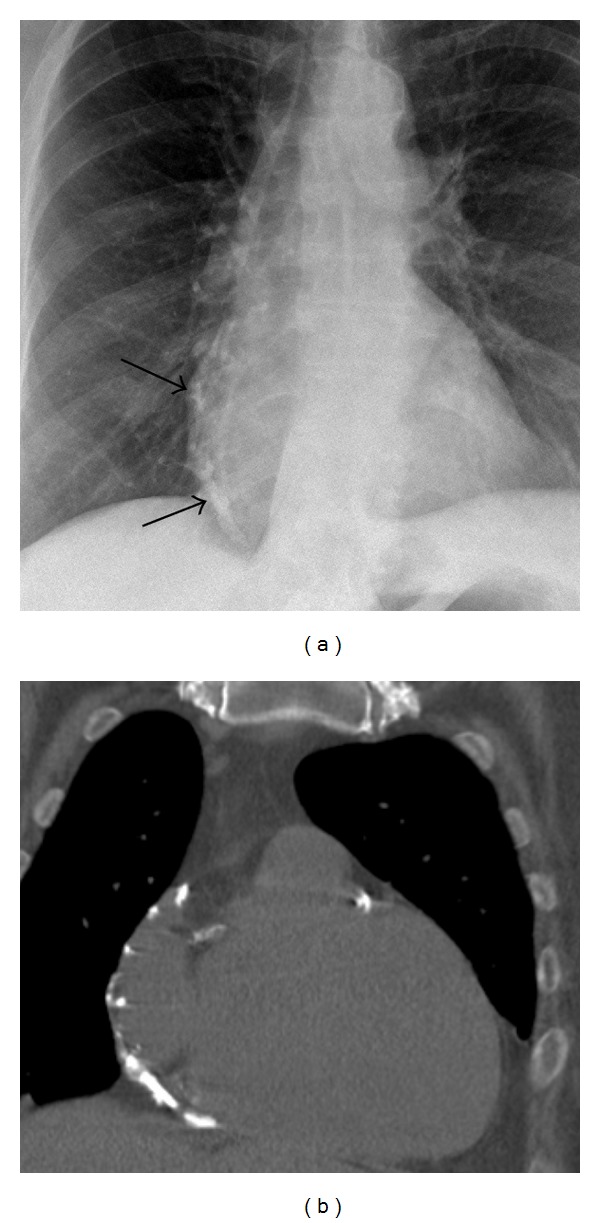
A 53-year-old woman with extensive cardiac history. (a) Chest radiograph and (b) coronal reconstructed CT image demonstrate dense calcification of the pericardium, predominantly on the right. With history of myocardial infarction, pericarditis, systemic lupus erythematosus, and end-stage renal disease, she has several independent possible explanations for her pericardial calcifications.

Noncompliance of the pericardium that results in impaired cardiac function is called constriction [24]. Tamponade and constriction may both lead to the phenomenon of “ventricular interdependence” (refer to explanation in the Section 4) [4]. Furthermore, distinguishing pericardial constriction from restrictive cardiomyopathy can be a diagnostic challenge but is clinically important, as the former is often treatable surgically, but the latter is not [8]. Pericardial calcification is most reliably demonstrated on CT [25]. While pericardial thickening and calcification are findings associated with constriction, they are not always be present (Figure 4). About 50% of patients with pericardial calcification will have constrictive physiology, and about 90% of patients with constrictive physiology will have pericardial calcification [17]. In addition, up to 20% of patients with constriction physiology have no significant pericardial thickening [25, 26]. Pericardial thickening may also be limited to only one portion of the pericardium. If this area of thickening is not included in the field-of-view of echocardiography, a false negative result can occur [8].

**Figure 5:**
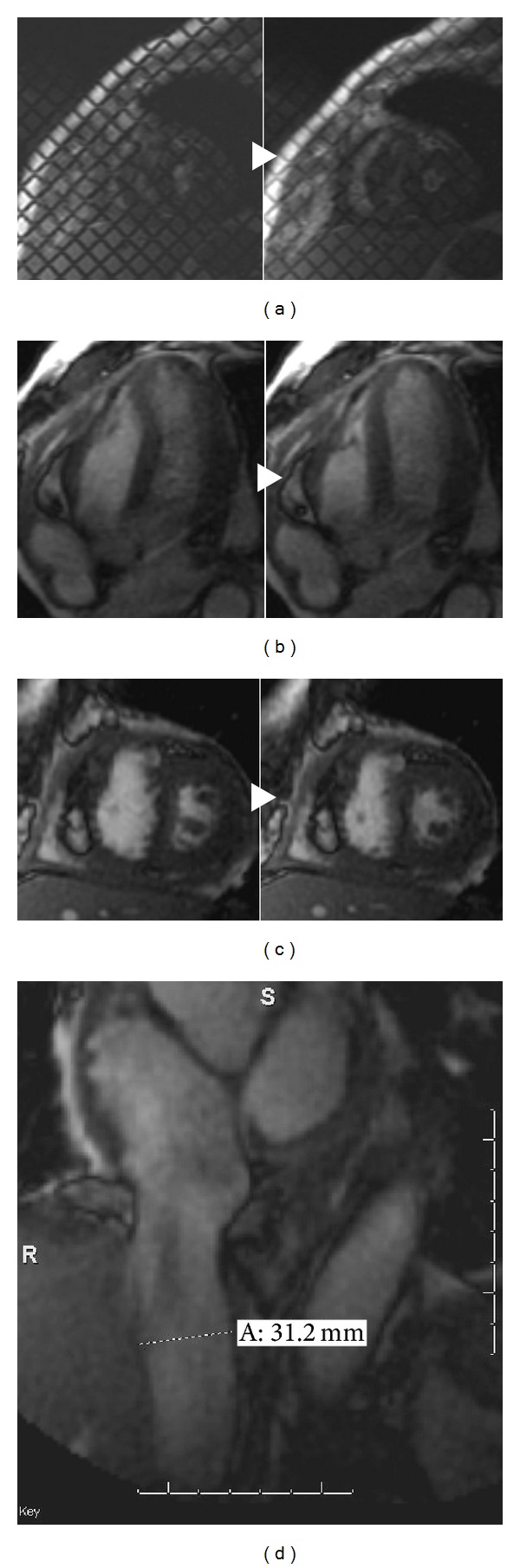
Constrictive pericarditis. A 67-year-old male with seropositive rheumatoid arthritis. An echocardiogram (images not available) was performed showing pericardial thickening and a small effusion. Paired images a, b, and c represent static images from dynamic MR imaging sequences. Cine tagged MR imaging: Transient fiducial grid-patterned image markers on sagittal images (a) demonstrates failure to dephase after several seconds, indicating nonslippage. Had the pericardium moved with respect to myocardium, the tag lines would have been broken. Instead they deformed only slightly, indicative of pericardial adhesion and providing evidence for constriction. (b, c) Breath-held SSFP long and short axis images demonstrate “septal bounce.” (d) The inferior vena cava is distended at 3.1 cm, providing corroborating evidence for elevated right heart pressures [30].

MR techniques have emerged which surpass both CT and echocardiography in the diagnosis of pericardial constriction. Morphology is assessed by measuring thickness of the entire pericardium in multiple planes. Function is evaluated by assessing pericardial motion in relation to myocardial motion, typically via steady-state free precession sequences, in combination with cine tagged imaging [16]. In the latter, a transient fiducial linear orthogonal grid pattern is generated by the pulse sequence; the resulting lines referred to as “tag lines.” Lack of normal pericardial “slippage” (i.e., adherence) is inferred when the tag lines fail to dephase (remain unbroken) (Figure 5(a)) [8, 27].

The impact of pericardial function on myocardial motion can be inferred by observing motion of the interventricular septum during MR cine imaging [28]. Flattening or convexity of the interventricular septum toward the left in early diastole indicates elevated right ventricular pressures. Later in diastole, LV pressure overcomes the elevated RV pressure. On cine imaging this resembles a rocking motion of the septum (“septal bounce”) indicative of ventricular interdependence (Figures 5(b) and 5(c)) [7]. The combination of pericardial nonslippage and ventricular interdependence is suggestive of pericardial constriction [4]. Engorgement of the inferior vena cava and hepatic veins may provide corroborative evidence for elevated right heart pressures (Figure 5(d)) [17, 29].

## 6. Inflammation

**Figure 6:**
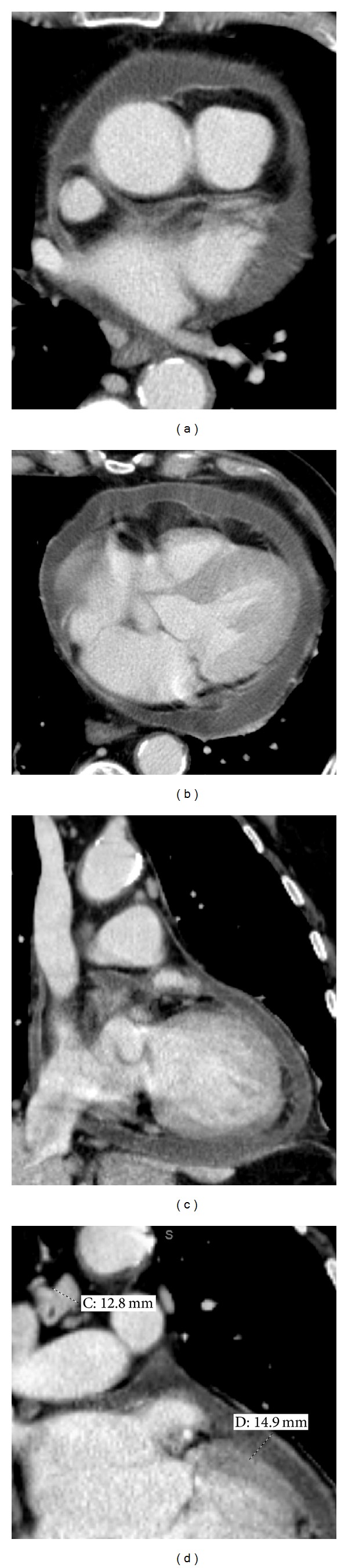
Idiopathic/viral pericarditis. An 83-year-old male with history of coronary artery disease and hypertension presents to the emergency department with a 5-week history of fever and malaise. CECT performed for fever of unknown origin demonstrates pericardial enhancement and effusion (a–c), mediastinal lymphadenopathy (d), and a solid enhancing right renal mass (not shown). No cause for the patient's pericarditis was found. Aspiration yielded occasional lymphocytes. Culture was negative. Fine needle aspiration of a mediastinal lymph node showed reactive cells. Symptoms gradually resolved on aspirin 325 mg daily. The patient's incidentally discovered that renal cell carcinoma proved to be nonmetastatic by PET-CT which was performed later.

Nonsuppurative pericarditis may be acute, chronic or recurrent. In otherwise healthy patients, pericarditis is often ascribed to an undiagnosed viral infection (Figure 6). In patients who have received in excess of 40 Gy of radiation to the chest (most commonly in the treatment of breast cancer or lymphoma), a sterile pericarditis may develop several months after the initiation of treatment [25]. In patients with autoimmune or collagen vascular diseases, any of the serosal surfaces of the body may become inflamed, and the pericardium is no exception. When pericarditis is chronic or recurrent in these patients, fibrosis may develop, resulting in constrictive physiology. Findings on imaging include pericardial thickening, effusion, calcification, or a combination of these. MRI is often performed to differentiate pericarditis from myocarditis, but both may be present [30]. Although the clinical presentation of pericarditis and myocarditis may be similar, myocardial involvement portends a longer duration of illness and greater risk of cardiac dysfunction or death.

## 7. Infection

**Figure 7:**
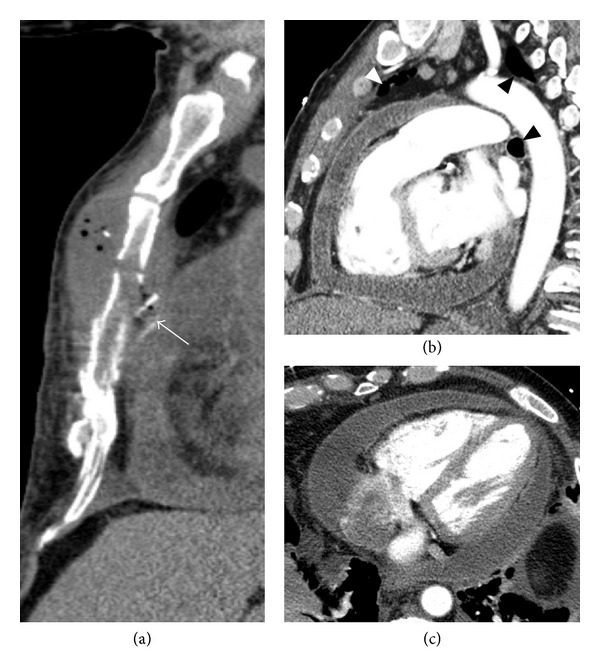
A 59-year-old diabetic male presents to the emergency department complaining of worsening dyspnea 5 weeks after sustaining a fractured sternum in a motor vehicle collision. Initial noncontrast CT (a) demonstrates a gas-containing collection at the site of the sternal fracture and a bone fragment projecting posteriorly (arrow). CECT ((b) sagittal and (c) axial) shows pericardial hyperenhancement and effusion. Mediastinal gas is present (arrowheads). Separate aspirations of the sternal collection and pericardial fluid both yielded pus which grew methicillin-sensitive *Staph. aureus*. Broad-spectrum antibiotic therapy was begun and a pericardial window was placed.

The most common pericardial infection is viral, but bacterial, fungal, and atypical infections may occur, particularly in the setting of penetrating trauma, the postpericardiotomy period, immunosuppression, and sepsis. Tuberculous and fungal organisms cause chronic infections in immunosuppressed patients, usually leading to constrictive disease [21]. The most common bacterial pathogens are *Staphylococcus, Streptococcus, Haemophilus*, *Propionibacterium*, and* Mycoplasma* (Figure 7) [31]. Anaerobes may involve the pericardium by fistulization from the GI tract.

## 8. Cyst

**Figure 8:**
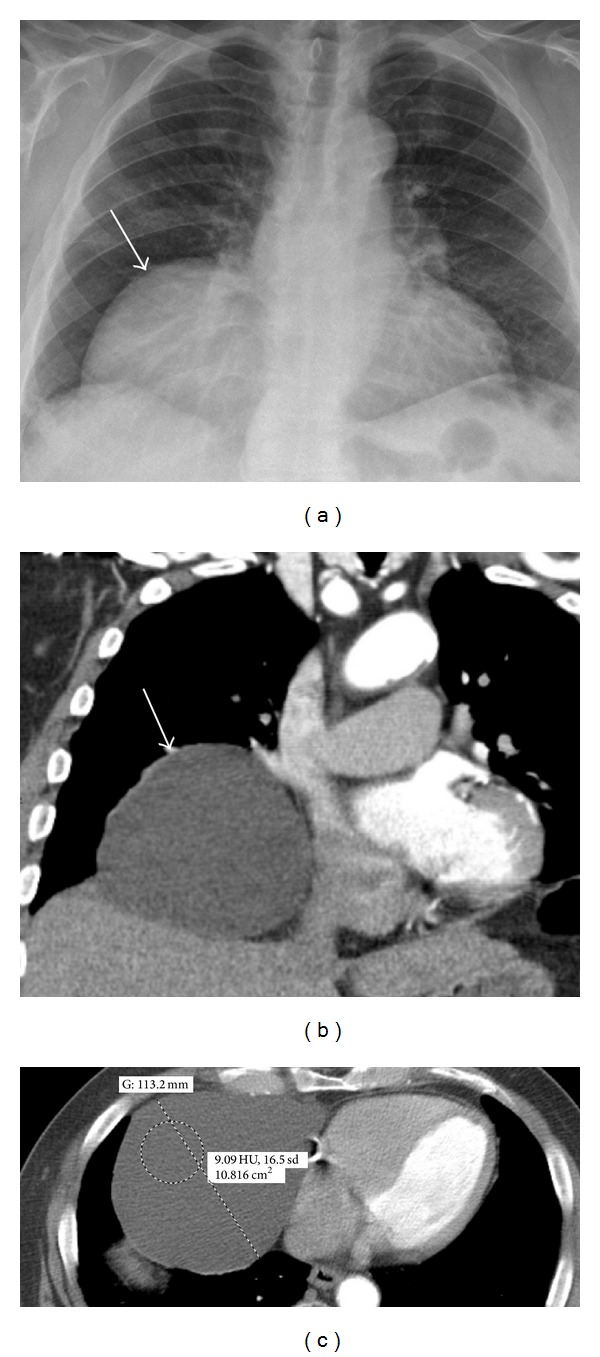
A 77-year-old male with shortness of breath. Chest radiograph (a) demonstrates an abnormal cardiac silhouette with prominence of the right cardiac border (arrow). The subsequent CECT (b, c) demonstrates an 11 cm pericardial cyst (arrow) containing simple fluid (HU of 9) without mass effect upon the SVC or IVC. Dyspnea was more likely related to early emphysematous and interstitial pulmonary changes demonstrated on the CT (not shown here).

Pericardial cysts are considered to be congenital but may enlarge over time [32]. The most common locations are right cardiophrenic angle (70% of cases), left cardiophrenic angle (20%), superior mediastinum (5%), and posterior mediastinum (5%) [33]. Pericardial cysts are usually incidental findings found on chest radiography or chest CT performed for other reasons (Figure 8). In rare cases, there may be signs and symptoms of mass effect, and resection may be considered in these cases [32]. Alternative differential considerations for a cystic structure in the region of the pericardium include foregut duplication cyst, neurenteric cyst, eventration of the diaphragm, Morgagni hernia, thoracic pancreatic pseudocyst, cystic neoplasm (lymphangioma, and hemangioma), and hydatid cyst.

## 9. Primary Neoplasm

**Figure 9:**
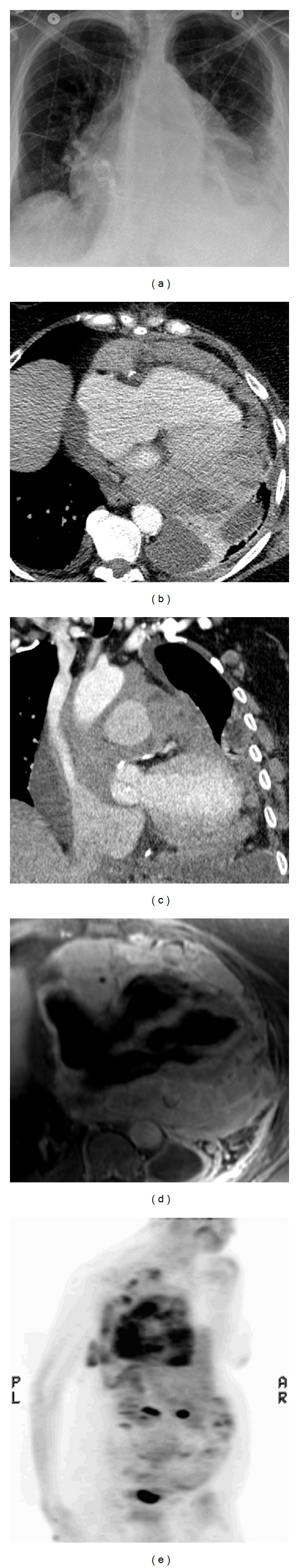
A 73-year-old female with prior history of breast cancer. She developed exertional dyspnea, which was found to be due to a pericardial effusion. This was treated semiemergently by pericardial window. Subsequent CECT showed progressive nodular pericardial thickening (b, c), as well as marked enhancement on MR (d). Planar FDG-PET image (e) shows markedly elevated pericardial metabolic activity and left pleural metastases. This was presumed to represent recurrent breast cancer presenting as pericardial metastatic disease, but biopsies returned malignant epithelioid mesothelioma.

Malignancy may involve the pericardium in three ways: primary neoplasm, metastasis, and direct invasion (most commonly by lung cancer). Of these, primary neoplasm is the least common [34, 35]. Benign pericardial neoplasms include fibroma, lipoma, hemangioma, and teratoma. Malignant histologies include mesothelioma, sarcoma, and lymphoma (Figure 9) [36].

Symptoms related to neoplastic involvement of the pericardium are often mild, due to the long period of time typically required for pericardial masses and malignant effusions to enlarge [37]. When acutely symptomatic, two distinct physiologic effects may occur. Constrictive physiology or tamponade physiology producing ventricular interdependence can occur [33]. Alternatively, compression of the systemic and/or pulmonary veins may lead to reduced preload to the right and left heart, respectively. The distinction between these two phenomena may be of little clinical importance, as palliation is easily performed via a pericardial window procedure (usually via a subxiphoid approach), or via intrapericardial instillation of a sclerosing agent or both [38, 39].

## 10. Metastasis

**Figure 10:**
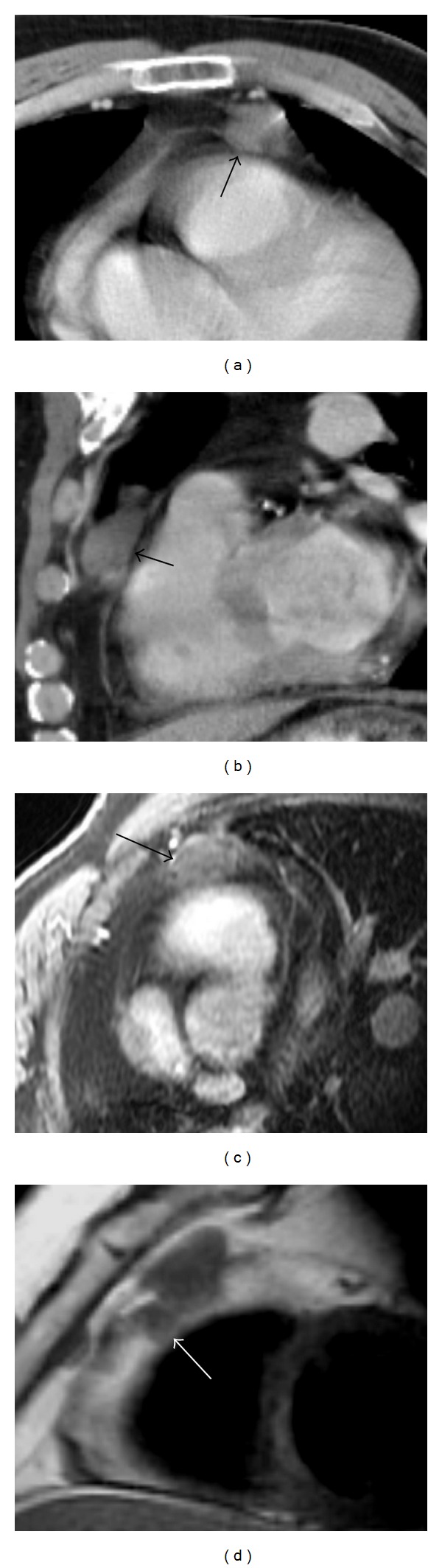
A 63-year-old male with cutaneous T-cell lymphoma, refractory to all modalities including total body electron irradiation. Axial (a) and sagittal (b) CECT images show a mass situated over the right ventricle, appearing to arise from the anterior pericardium (arrows). (c) On spoiled gradient postcontrast axial MR the mass enhances homogeneously and straddles the pericardium (arrow). (d) Double inversion recovery sagittal image demonstrates that the mass has invaded through the pericardium into the epicardial fat (arrow).

Pericardial metastases are more common than suspected on clinical grounds, as they are found in 1.5 to 22% of autopsy specimens of patients who died from cancer [40]. In other words, patients usually die of their disease before the pericardial metastases become physiologically important. The most common primary malignancies with pericardial metastases are breast, lung, lymphoma and melanoma, but any widely metastatic malignancy may implant on the pericardium (Figure 10) [4]. Metastasis to the pericardium occurs by both hematogenous and lymphatic routes. Pericardial metastatic disease may cause constriction by encasement of the heart. Alternatively, it may impair cardiac function via malignant effusion and tamponade physiology [33]. More commonly, as with primary pericardial neoplasm, symptoms are insidious. Imaging findings typically include nodular pericardial thickening with enhancement and effusion [19, 35]. These are of course non-specific findings and definitive diagnosis can be difficult without biopsy [41].

## 11. Conclusion

The pericardium can be affected by a variety of pathologies with important physiologic consequences. In acute pericardial dysfunction from rapid pericardial fluid accumulation (i.e., tamponade), death may occur rapidly in the absence of intervention. In more chronic conditions, pericardial dysfunction from constriction can be treated surgically via resection or window placement. Echocardiography remains an important first-line imaging modality in the evaluation of pericardial disease, particularly in the acute setting at the bedside. The development of multidetector CT and cardiac MR pulse sequences has improved the ability of diagnostic imagers to evaluate pericardial disease and dysfunction on static and dynamic imaging, allowing more timely and appropriate treatment.

## Figures and Tables

**Table 1:** MR techniques commonly used to evaluate the pericardium.

MR technique	Clinical utility for pericardium
Steady-state free precession (SSFP).	Wall motion, especially septum; effusion.
Double inversion recovery fast spin echo (DIR FSE).	Pericardial thickness; effusion.
T1-weighted gradient echo myocardial tagged cine.	Presence or absence of epicardial-pericardial slippage.
Real-time cine GRE in short axis during dynamic breathing.	Assess for ventricular interdependence.
Optional: T2 FSE, T1/T2 fat saturation; postcontrast imaging.	Used when tissue characterization is important, for example, neoplasm.

**Table 2:** Abbreviated differential diagnosis of pericardial effusion [18, 42].

Disease category	Disease entity
Systemic hemodynamic	Congestive heart failure
	Post-MI (Dressler) syndrome
	Cirrhosis

Metabolic	Malnutrition
	Hypoalbuminemia
	Uremia
	Chronic hypothyroidism (myxedema)

Inflammatory/autoimmune	Rheumatoid arthritis
	Systemic lupus erythematosis
	Other connective tissue diseases
	Sarcoidosis
	Sympathetic effusion due to sepsis

Infectious	Viral
	Suppurative (bacterial)
	Tuberculous
	Fungal
	Parasitic

Traumatic/iatrogenic	Chronic traumatic hematoma
	Postpericardiotomy syndrome
	Radiation pericarditis
	Postcardiac surgery/intervention

Neoplastic	Metastasis
	Primary neoplasm
	Lymphoma

Other	Drug reaction
	Chylopericardium
	Idiopathic
